# Supplementary material for: Spatiotemporal dynamics of local anesthetic diffusion in nerve revealed by a 2D computational model
Source: Biophys J. 2025 Sep 19;124(21):3706–16. doi: 10.1016/j.bpj.2025.09.026 (PMC12709419; doi:10.1016/j.bpj.2025.09.026)
Supplement: Document S1. Figures S1 and S2 [file mmc1.pdf]

Biophysical Journal, Volume *124*

Supplemental information

Spatiotemporal dynamics of local anesthetic diffusion in nerve revealed by a 2D computational model

Vladimir Smrkolj, Jakob Kralj, Janez Mavri, and Nejc Umek

Spatiotemporal dynamics of local anesthetic diffusion in nerve revealed by a 2d computational model – Supplementary information

Vladimir Smrkolj[1,2], Jakob Kralj[2], Janez Mavri[2], Nejc Umek[1]

1 Institute of Anatomy, Faculty of Medicine, University of Ljubljana, Slovenia

2 Laboratory of Computational Biochemistry and Drug Design, National Institute of Chemistry, Ljubljana, Slovenia

Derivation of the replacement coefficient

For simplicity, let's examine the transfer between two adjacent cells:

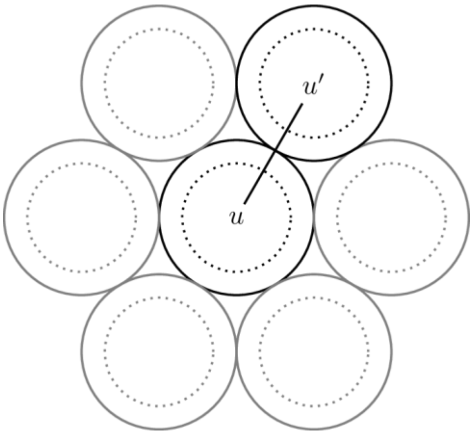

Figure S 1 Scheme of the compartment and it's soundings

Both cells have identical radius r, and may or may not be separated by a membrane. The transfer between these two compartments can be expressed as follows:

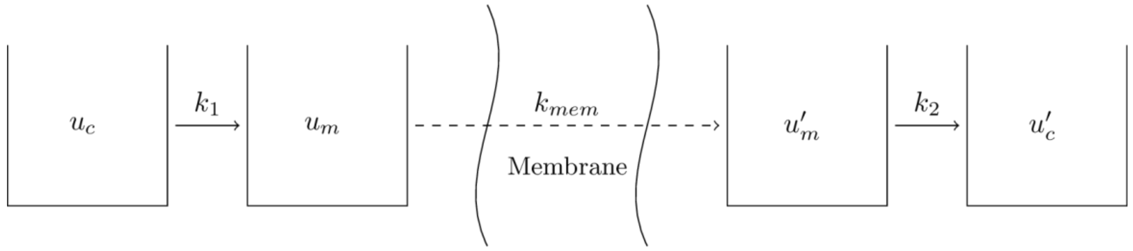

Figure S 2 Scheme of the transfer through the membrane

where u_c, u_m, u_m', u_c' represent compartments and k_1, k_2, k_{mem} denote rate constants. For the constants k_1 and k_2 we apply a standard approximation based on the diffusion coefficient D and cellular geometry:

$$k = \frac{2D}{r^2}$$

The membrane constant k_{mem} is derived from numerical simulations of the Smoluchowski PDE and varies depending on which two cells are adjacent (the rate constant differs when diffusing from inside to outside the neuron versus other directions). When diffusing between two compartments containing only inter-cellular fluid, no membrane exists, making the rate constant effectively infinite.

Considering the steady state solution, we see that the flow rate through the four compartments would be

$$J = k_1(u_c - u_m) = k_{\mathrm{mem}}(u_m - u_m') = k_2(u_m' - u_c')$$

Let's denote a replacement constant, so that

$$J = k^*(u_c - u_c').$$

Solving the system of equation yields:

$$\frac{1}{k^*} = \frac{1}{k_1} + \frac{1}{k_2} + \frac{1}{k_{mem}}.$$

Note that where no membrane is present, $k_{mem} = \infty$ and the last term becomes zero. The combined rate constant then becomes:

$$\frac{1}{k^*} = \frac{r^2 + r'^2}{2D} + \frac{1}{k_{mem}}$$

$$\frac{1}{k^*} = \frac{k_{mem}(r^2 + r'^2) + 2D}{2Dk_{mem}}$$

This yields the final replacement coefficient:

$$k^* = \frac{2Dk_{mem}}{k_{mem}(r^2 + r'^2) + 2D}$$
