# Supplementary material for: Spatiotemporal dynamics of local anesthetic diffusion in nerve revealed by a 2D computational model
Source: Biophys J. 2025 Sep 19;124(21):3706–16. doi: 10.1016/j.bpj.2025.09.026 (PMC12709419; doi:10.1016/j.bpj.2025.09.026)
Supplement: Document S2. Article plus supporting material [file mmc2.pdf]

Biophysical Journal
Article

Biophysical Society

Spatiotemporal dynamics of local anesthetic diffusion in nerve revealed by a 2D computational model

Vladimir Smrkolj,[1,2] Jakob Kralj,[2] Janez Mavri,[2] and Nejc Umek[1,*]
[1]Institute of Anatomy, Faculty of Medicine, University of Ljubljana, Ljubljana, Slovenia and [2]Laboratory of Computational Biochemistry and Drug Design, National Institute of Chemistry, Ljubljana, Slovenia

ABSTRACT Despite their extensive clinical use, the intraneural pharmacokinetics of local anesthetics, including the mechanisms determining their onset and duration, remain incompletely understood, particularly under pathological conditions. We developed a detailed, spatially compartmentalized computational model of human peripheral nerve fascicles to simulate the diffusion, accumulation, and clearance kinetics of lidocaine and bupivacaine under physiological and acidic conditions. The model integrates nerve fiber architecture, extracellular fluid compartments, and capillary-mediated clearance, parameterized using experimentally validated anatomical and physicochemical data. It was implemented in Python 3.12, employing a fourth-order Runge-Kutta integrator via the SciPy library. The results revealed that onset is limited primarily by extracellular diffusion rather than transmembrane transport. Lidocaine reached the predefined onset threshold ($\geq$50% of nerve fibers with $\geq$50% external concentration) at 8.7 s under physiological pH and 3.2 s under acidic conditions. Bupivacaine exhibited longer onset times, 79.2 s and 16.1 s, respectively. Acidic conditions markedly reduced equilibrium concentrations within nerve fibers (by 3.6-fold for lidocaine, 3.5-fold for bupivacaine), significantly shortening their durations of action (lidocaine: 758 to 233 s; bupivacaine: 6980 to 2059 s). These findings mirror known clinical efficacy reductions in inflamed or acidotic tissues. In conclusion, local anesthetic onset is primarily governed by extracellular diffusion rather than membrane permeability, rendering it largely independent of drug pKa. Local tissue acidosis reduces nerve fiber accumulation and accelerates anesthetic washout, thereby diminishing both efficacy and duration of action. These results explain long-standing clinical observations and suggest that nerve fibers act as kinetic reservoirs modulated by tissue pH. This computational model offers a valuable framework for predicting anesthetic behavior and optimizing drug delivery strategies in regional anesthesia, particularly under pathological conditions such as inflammation.

SIGNIFICANCE The effectiveness of local anesthetics is known to decline in inflamed or acidic tissues, but the underlying mechanisms have remained poorly understood. We developed a detailed computational model that simulates the diffusion and clearance of lidocaine and bupivacaine from human peripheral nerves under normal and acidic conditions. Our results show that onset time is primarily governed by extracellular diffusion—not membrane permeability—and that acidosis accelerates washout while reducing anesthetic accumulation. This work explains long-standing clinical observations and provides a mechanistic framework to improve local anesthetic design and delivery.

INTRODUCTION

Local anesthetics (LAs) are indispensable in modern medicine, enabling painless surgical and diagnostic procedures through the reversible inhibition of nerve conduction (1–4). However, despite their widespread use, the pharmacokinetics of LAs—particularly their distribution within nerve tissue—remains incompletely understood (1,2,5,6). Clinically observed variability in LA onset and duration is well documented and attributed to patient-specific factors, anatomical variations, and local tissue conditions (7–13).

LAs work by inhibiting voltage-gated sodium channels in neuronal membranes (2,14). Their efficacy depends on physicochemical properties, such as pK_a and lipophilicity, and pharmacodynamic properties, such as potency.

Submitted May 14, 2025, and accepted for publication September 17, 2025.
*Correspondence: nejc.umek@mf.uni-lj.si
Editor: Alex Mogilner.
https://doi.org/10.1016/j.bpj.2025.09.026
© 2025 The Author(s). Published by Elsevier Inc. on behalf of Biophysical Society.
This is an open access article under the CC BY license (http://creativecommons.org/licenses/by/4.0/).

Traditionally, pK_a and lipophilicity have been assumed to govern pH-dependent membrane permeability; however, recent studies (5,15,16), including ours (17), demonstrate that membrane permeation does not constitute the rate-limiting step in LA diffusion from the application site to the target. Although diminished LA efficacy in acidic environments (e.g., inflamed tissues) is frequently observed clinically, a quantitative understanding of these pH-dependent effects is still lacking (10,18,19). A mechanistic understanding of LA diffusion and retention within peripheral nerves is, therefore, crucial for optimizing their clinical use.

The aim of this study was to investigate the pharmacokinetics of lidocaine and bupivacaine after bolus application, using an in silico model simulating the spatiotemporal behavior of LAs within a human peripheral nerve model. By conducting parallel simulations under physiological and acidotic extracellular pH, we investigated how anesthetic physicochemical properties and tissue acidity influence their distribution dynamics.

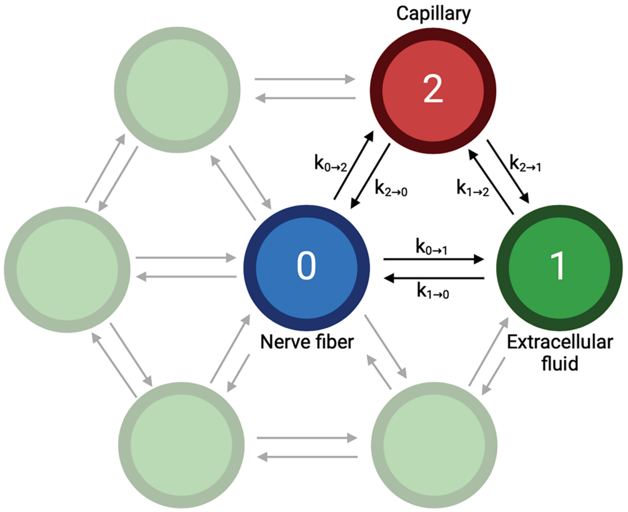

FIGURE 1 Simplified compartmental schematic used in the model. Uniform arrangement of one nerve fiber, five extracellular compartments, and one capillary. Three compartments are labeled with numbers for clarity. Figure was created with BioRender.com.

MATERIALS AND METHODS

Modeling local anesthetic diffusion requires numerically solving the Smoluchowski equation on two- or three-dimensional spatial grids (20). However, these numerical solutions are computationally intensive, primarily due to the small integration time steps required for stability and accuracy. Analogous to time-dependent Schrödinger equation simulations, adding spatial dimensions demands finer grids, which expand the spectral bandwidth and thus further restrict allowable time steps (21). To incorporate essential nerve anatomy and maintain simulation durations compatible with clinical timescales, we implemented an alternative, more computationally efficient modeling strategy.

We constructed a two-dimensional cross-sectional model of the human peripheral nerve as a network of compartments governed by ordinary differential equations (ODEs). These ODEs were solved using a fourth-order Runge-Kutta integrator implemented in Python 3.12, leveraging the SciPy library (22). The solver determines the step size automatically, but it was bounded to the maximum step of 0.001 s. Full model code is freely available at https://github.com/ladismrkolj/2d-ode-diffusion. The model includes key anatomical structures such as nerve fibers, extracellular fluid, and capillaries within the fascicle (23). Each compartment is represented as a node, with the ODEs defining LA flux between connected nodes (Fig. 1).

The anatomical dimensions and node arrangements were derived from experimental and imaging studies. In a typical nerve fascicle, the density of nerve fibers is 25,969 mm^{-2} (24), and the ratio of cross sections of nerve fibers to extracellular tissue is 3:2 (24,25). In addition, the density of capillaries in human myelinated fascicles is 67.9 mm^{-2} (26).

Neighboring compartments were coupled via kinetic terms in the ODEs, accounting for bidirectional LA transfer. Intercompartmental rate constants were derived from our previous one-dimensional Smoluchowski equation-based model of LA transfer within nerve fascicle tissues (17). These rate constants were further adjusted to incorporate intracompartmental diffusion times, reflecting the transit time within each compartment. Specifically, LA transfer between the centers of two adjacent compartments involves three sequential steps: 1) diffusion from the center of the first compartment to the intervening membrane, 2) permeation through the membrane, and 3) diffusion from the membrane to the center of the second compartment. This three-step process is mathematically equivalent to a system of four effective states (or compartments) connected by three sequential rate constants. The three rate constants can be combined into a substitute rate constant, using a principle termed "pharmacological lumping" (27). The substitute rate constant (k^*) can be calculated from the three rate constants as

$$\frac{1}{k^*} = \frac{1}{k_1} + \frac{1}{k_2} + \frac{1}{k_3}, \tag{1}$$

where k_1 is the rate constant for diffusion from the center of the first compartment to the membrane, k_2 is the rate constant for membrane crossing, and k_3 is the rate constant for diffusion from the membrane to the center of the second compartment. We assumed that pathways involving multiple or nonadjacent compartments contribute negligibly to the overall LA flux, and that any such minor effects are effectively subsumed within the parameterization of Eq. (1). In a solution with a diffusion coefficient of D, the mean first passage time, t, of a one-dimensional travel of a distance x is $t = \frac{x^2}{2D}$. Although our simulation is two-dimensional, only the normal component of the travel to the membrane is relevant, so it becomes a one-dimensional problem. The rate constant is the inverse of this and is expressed as $k = \frac{2D}{x^2}$.

We can express the adjusted rate constant (k^*) by substituting the rate constants in Eq. 1 with the rate constant of transfer through the lipid membrane between the compartments (k) and the rate constants of diffusion through distances x_1 and x_2, which represent the distances from the center of each neighboring compartment to the membrane:

$$\frac{1}{k^*} = \frac{x_1^2}{2D} + \frac{1}{k} + \frac{x_2^2}{2D} = \frac{x_1^2 \cdot k + 2D + x_2^2 \cdot k}{2D \cdot k} = \frac{\left(x_1^2 + x_2^2\right) \cdot k + 2D}{2D \cdot k} \tag{2}$$

Finally, the substitution rate constant is

$$k^* = \frac{2D \cdot k}{\left(x_1^2 + x_2^2\right) \cdot k + 2D} \tag{3}$$

A detailed derivation of the compartmental rate constants is provided in the supporting material.

The rate constant between two extracellular fluid compartments is not dependent on any rate constant of transfer through the lipid membrane; therefore, it is expressed only in terms of the distance between the centers of the two compartments (x):

$$k = \frac{2D}{x^2} \tag{4}$$

Capillary compartments were treated as extracellular fluid compartments with an additional negative term describing capillary clearance. As LA transfer into capillaries is considered perfusion limited, more complex clearance models were deemed unnecessary (28). In this model, the capillary clearance rate is equated to local blood flow, which is determined from the capillary blood velocity and cross-sectional area. Using 0.79 mm s^{-1} (29) as the blood velocity in human capillaries and a cross section of a typical capillary of 33 μm^2 (26), the blood flow is calculated to be 2.61×10^{-5} $\mu L\ s^{-1}$. Furthermore, this basal blood flow rate was adjusted to account for vasomotion, as approximately 25% of capillaries are understood to be transiently closed at any given moment. This physiological consideration reduces the effective blood flow per capillary to 1.96×10^{-5} $\mu L\ s^{-1}$.

Boundary conditions for the model were defined for two distinct operational phases: an initial "filling" phase and a subsequent "voiding" phase. During the filling phase, which simulates the influx of LA into the nerve fascicle immediately after bolus application, Dirichlet boundary conditions were applied. This involved setting the LA concentration at the external boundary of the modeled fascicle to a constant, normalized value of 1, representing complete immersion in an LA solution of unity concentration. During the voiding phase, simulating LA efflux through capillaries, Neumann boundary conditions were applied; this choice reflects simulating a single nerve fascicle within a larger, periodically arranged nerve structure, implying zero flux across fascicle boundaries.

Finally, the complete input data for the model were defined. These include the pattern of nerve fibers and capillaries on a two-dimensional grid, the rate constant of permeation of LAs from the extracellular fluid into the nerve fiber ($k_{E \to N}$), the rate constant of permeation of LAs from the nerve fiber into the extracellular fluid ($k_{N \to E}$), clearance through the capillary, and the diffusion coefficient in water (D) for each local anesthetic. For the nerve fiber pattern, a hexagonal geometry was selected to facilitate code optimization, although our model accommodates arbitrary input geometry. Fig. 2 shows the hexagonal pattern of 1254 nerve fiber compartments, seven capillary compartments and 3799 extracellular fluid compartments. The diameter of this pattern is 140 μm.

Rate constants were adopted from our prior micropharmacokinetic analysis of intrafascicular local anesthetic transport (17) and normalized to each compartment's volume, which is computed at the start of each simulation based on the defined geometric arrangement. The rate constants are from the area of the Schwann cell. Diffusion coefficients for lidocaine and bupivacaine were obtained from Brounéus et al. (30). Values for the rate constants and diffusion coefficients for lidocaine and bupivacaine under physiological or acidotic conditions are presented in Table 1. The capillary clearance is 1.96×10^{-5} $\mu L\ s^{-1}$, as previously calculated.

Space- and time-dependent LA concentration profiles were quantitatively analyzed to determine onset time and duration of action for both local anesthetics under different conditions. Given that the minimal inhibitory concentration of LAs at the site of action is unknown, we assumed the effect is present when >50% of nerve fibers achieve a concentration >50% of the initial external concentration (31). Therefore, onset time was defined as the simulation time elapsed until this threshold was first met. Similarly, duration of action was defined as the total continuous time interval during which this criterion remained satisfied. This time was also annotated as $t_{1/2}$.

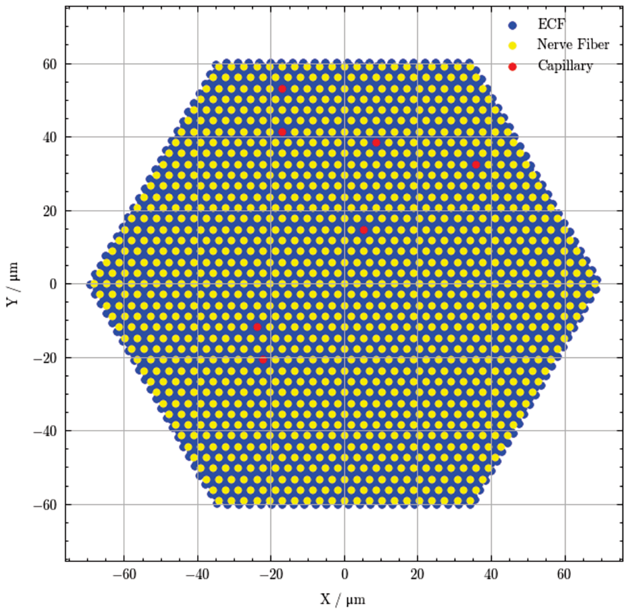

FIGURE 2 Hexagonal lattice representation of a peripheral nerve fascicle. Uniform hexagonal arrangement of 1254 nerve fiber compartments, seven capillary compartments, and associated extracellular fluid (ECF) compartments within a modeled fascicle (diameter: 140 μm). This arrangement represents the structural basis for simulating local anesthetic diffusion and clearance dynamics.

RESULTS

Space- and time-dependent concentration profiles

The primary output of the computational model consisted of spatiotemporal concentration profiles for each LA. These profiles were analyzed and are presented separately for two distinct phases: the "filling" phase (simulating LA influx) and the "voiding" phase (simulating LA efflux and clearance). For each LA (lidocaine and bupivacaine) and condition (physiological or acidotic pH), spatial concentration distributions within the nerve fascicle are depicted at four representative time points. These distributions are color-coded, and a consistent concentration scale is applied across each series of profiles to facilitate direct comparison. Space- and time-dependent concentration profiles for lidocaine and bupivacaine during the filling phase under physiological and acidotic conditions are shown in Fig. 3.

LA diffusion within the nerve followed a clear spatial gradient, with concentrations increasing from the periphery toward the interior. Within nerve fibers, LA concentration exceeded the equilibrium level in the surrounding extracellular fluid (normalized to 1), creating a honeycomb-like profile where peripheral compartments achieved equilibrium faster than central compartments. Notably, equilibrium LA levels were substantially higher in the hydrophobic nerve fiber compartments than in the extracellular fluid.

TABLE 1 Values of rate constants and diffusion coefficients for lidocaine and bupivacaine under physiological or acidic conditions

Local Anesthetic – condition	Diffusion coefficient (D)/m^{-2} s^{-1}	$k_{E \rightarrow N}$/μl s^{-1}	$k_{N \rightarrow E}$/μl s^{-1}
Lidocaine – physiological	$7.49 \cdot 10^{-10}$	$9.147 \cdot 10^{-3}$	$2.152 \cdot 10^{-4}$
Lidocaine – acidosis	$7.49 \cdot 10^{-10}$	$2.322 \cdot 10^{-2}$	$1.942 \cdot 10^{-3}$
Bupivacaine – physiological	$6.71 \cdot 10^{-10}$	$3.774 \cdot 10^{-3}$	$1.093 \cdot 10^{-5}$
Bupivacaine – acidosis	$6.71 \cdot 10^{-10}$	$6.948 \cdot 10^{-3}$	$7.127 \cdot 10^{-5}$

Acidic conditions markedly altered diffusion dynamics. Although equilibrium concentration within nerve fiber compartments was substantially reduced (3.6-fold for lidocaine, 3.5-fold for bupivacaine versus physiological conditions), the anesthetic spread radially within the fascicle cross section more rapidly. This accelerated diffusion under acidosis was evident as a broader band of lighter-colored compartments in the time series images.

For bupivacaine, which has a diffusion coefficient of 6.71×10^{-10} m^2/s (approximately 10.4% lower than that of lidocaine (7.49×10^{-10} m^2/s)), we extended the simulation time (with profiles at 0, 2, 10, and 20 s). As a result, bupivacaine penetrates the nerve more slowly than lidocaine does, as indicated by a thinner band of elevated concentration and a smaller proportion of nerve fiber compartments reaching final equilibrium at any given time. Despite these differences in kinetics, the overall pattern of faster diffusion under acidic conditions was consistent for both anesthetics.

Next, we examined the voiding phase of LA washout from the nerve. Fig. 4 displays simulated cross-sectional images over successive time points, illustrating the spatial distribution of lidocaine and bupivacaine under both physiological conditions and local acidosis.

The figure presents a series of cross-sectional snapshots from a hexagonally modeled nerve fascicle at multiple time points to illustrate the washout of both lidocaine and bupivacaine under physiological and acidic conditions. At $t = 0$ s, each anesthetic is distributed uniformly throughout the fascicle, as indicated by lighter color tones. Over time, the color transitions toward darker shades, signifying a reduction in anesthetic concentration.

Under all tested washout conditions, the LA concentration appeared to decrease in a relatively uniform manner across the fascicle, without the formation of pronounced low-concentration zones, for instance, immediately surrounding capillaries. Visual inspection of the concentration profiles indicated that, under acidotic conditions, both lidocaine and bupivacaine cleared more rapidly from the fascicle than under physiological pH. Furthermore, although bupivacaine achieved higher initial saturation concentrations within the fascicle compared with lidocaine (reflecting greater partitioning), its subsequent washout was visibly slower, as evidenced by the longer persistence of color indicators representing higher concentrations. By the final depicted time point in these washout simulations, fascicular LA concentrations were substantially reduced in all scenarios—represented by a predominant shift to color mapping indicative of low concentrations—signifying extensive clearance of the anesthetic from the tissue.

Time to onset and duration of action estimation

Onset time was estimated as the time required for >50% of nerve fiber compartments to reach >50% of the initial external anesthetic concentration. Fig. 5 presents the fraction of nerve fiber compartments surpassing this threshold and their average anesthetic concentration, for lidocaine and bupivacaine under physiological and acidic conditions.

For lidocaine, the proportion of nerve fiber compartments exceeding >50% of the initial external anesthetic concentration initially increases steeply before gradually slowing, exhibiting a curve characteristic of exponential growth approaching an asymptote (a theoretical maximum of 1, representing all compartments). Under acidic conditions, the onset threshold (>50% fiber fraction) is met significantly faster. The estimated onset time is 8.69 s under physiological conditions and 3.16 s under acidic conditions. Importantly, these calculated onset times depend on the fascicle dimensions used in the model; our fascicle model had a diameter of 120 μm, containing 1254 uniformly distributed nerve fibers and seven capillaries. These results align closely with the spatial and temporal concentration distributions illustrated previously. Although the number of compartments surpassing the threshold increases faster under acidic conditions, their overall mean anesthetic concentration remains lower than under physiological conditions, indicating that during acidosis, more compartments rapidly receive a smaller total quantity of anesthetic. Bupivacaine exhibited substantially slower onset kinetics, such that only the first 30 s of the simulation are shown for clarity. The onset times for bupivacaine were markedly longer: 79.2 s under physiological conditions and 16.1 s under acidotic conditions. Compared with lidocaine, bupivacaine's onset was prolonged by factors of 9.11 (physiological conditions) and 5.09 (acidotic conditions). However, similar to the case of lidocaine, more nerve compartments achieve the 50% external concentration threshold faster under local acidosis than under physiological conditions.

Duration of action estimation

Duration of anesthetic action was estimated as the time, starting from equilibrium, for concentration to drop below 50% of its initial equilibrium value in at least 50% of nerve

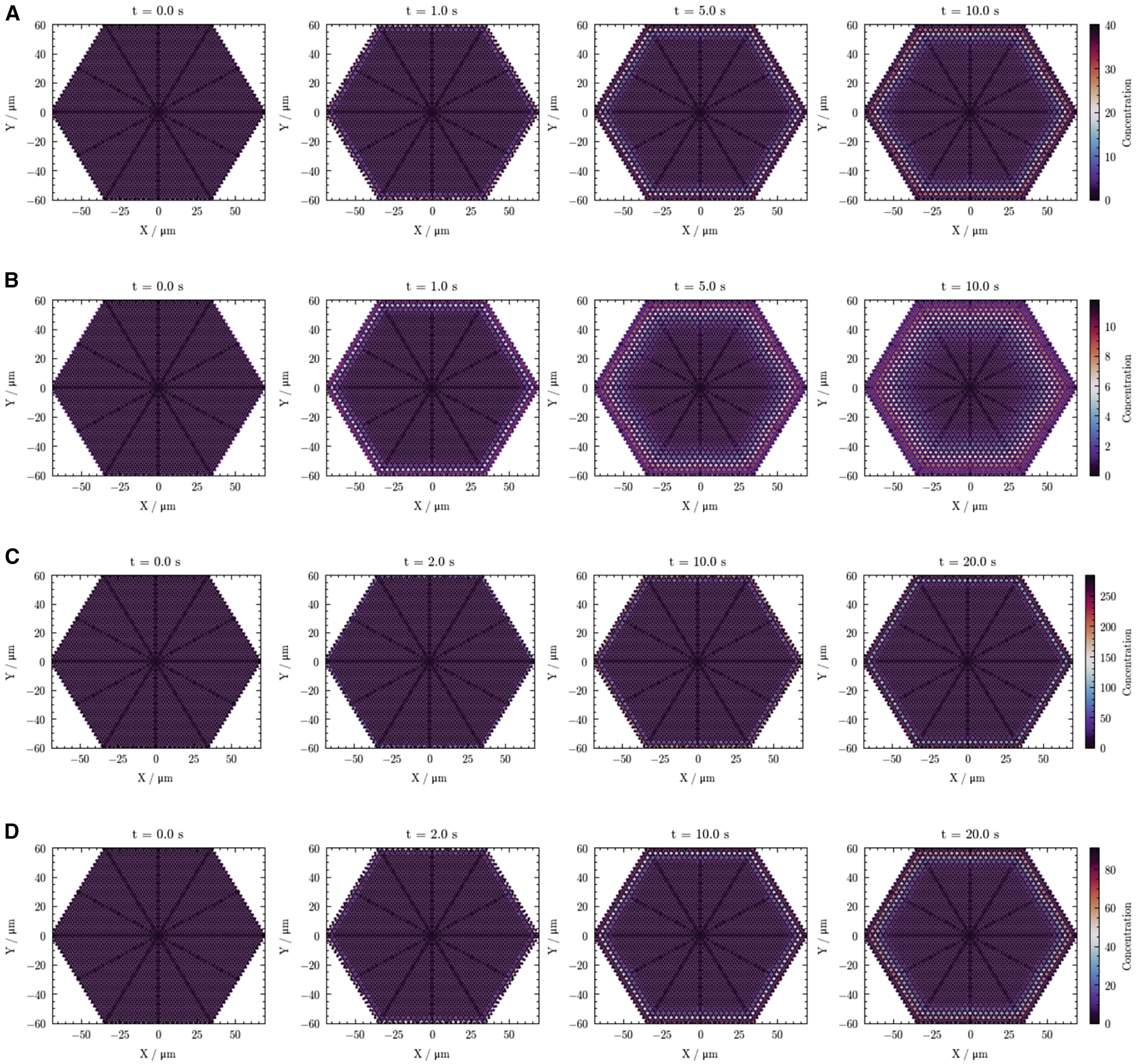

FIGURE 3 Filling-phase spatial concentration profiles for lidocaine and bupivacaine under physiological and acidotic pH. Panels show lidocaine under physiological conditions (*A*), lidocaine under local acidosis (*B*), bupivacaine under physiological conditions (*C*), and bupivacaine under local acidosis (*D*). Concentration distributions are shown at selected time points: 0 s, 1 s, 5 s, and 10 s for lidocaine and 0 s, 2 s, 10 s, and 20 s for bupivacaine. The color gradient represents the relative anesthetic concentration, with lighter colors indicating higher concentrations and darker colors indicating lower concentrations.

fiber compartments. Fig. 6 presents the fraction of compartments exceeding 50% of initial equilibrium concentration and their average anesthetic concentration over time, illustrating the washout dynamics of lidocaine and bupivacaine under physiological and acidic conditions.

Consistent with spatial analysis, anesthetic concentration declined uniformly within nerve fiber compartments. However, unlike the gradual increase during filling, the proportion of compartments retaining at least half of the initial equilibrium concentration decreased abruptly, reflecting a sudden widespread reduction in anesthetic levels. Correspondingly, the average concentration within nerve fiber compartments exhibited a continuous exponential decrease toward an asymptote near zero.

Under local acidosis, lidocaine's initial equilibrium concentration was approximately 3.6-fold lower than under physiological conditions. The estimated duration of action under physiological conditions was 758 s, whereas it was 233 s under acidic conditions, corresponding to a 3.25-fold shorter half-life during acidosis. The half-life, defined as the time when >50% of nerve fiber compartments drop below 50% of initial equilibrium concentration, closely

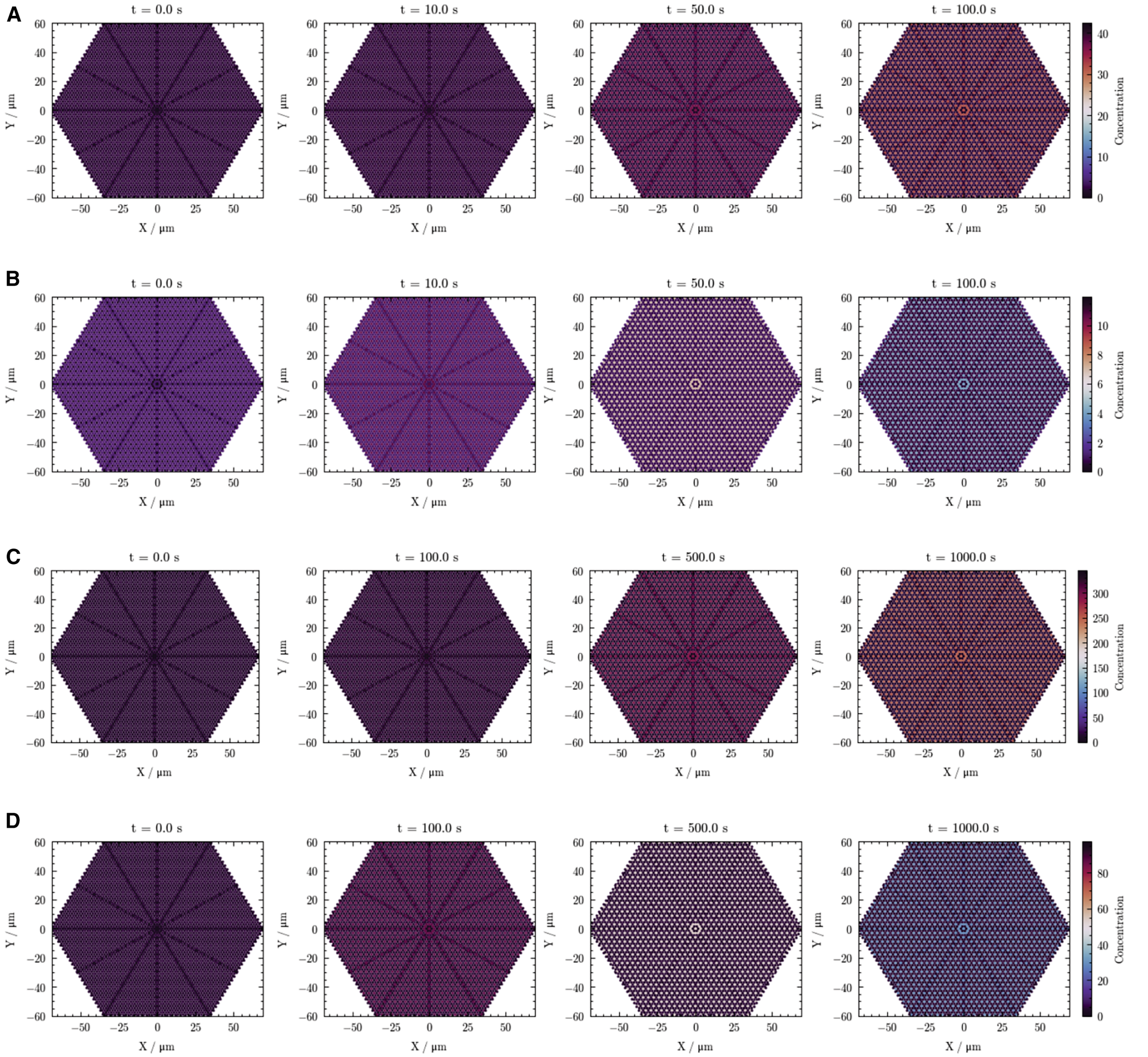

FIGURE 4 Voiding-phase spatial concentration maps illustrating washout kinetics of lidocaine and bupivacaine under physiological and acidotic pH. Panels show lidocaine under physiological conditions (*A*), lidocaine under local acidosis (*B*), bupivacaine under physiological conditions (*C*), and bupivacaine under local acidosis (*D*). Concentration distributions are shown at selected time points: 0 s, 10 s, 50 s, and 100 s for lidocaine and 0 s, 100 s, 500 s, and 1000 s for bupivacaine. The color gradient represents the relative anesthetic concentration, with lighter colors indicating higher concentrations and darker colors indicating lower concentrations.

aligns with the time for average concentration within nerve fiber compartments to reach half of its initial equilibrium. Similarly, bupivacaine's average concentration also displayed a continuous exponential reduction, asymptotically approaching zero. The initial equilibrium concentration under local acidosis (97.5) was approximately 3.5-fold lower than that under physiological conditions (345.3). Estimated duration of action for bupivacaine was substantially longer than for lidocaine: 6980 s (physiological) and 2059 s (acidic), representing a 3.39-fold reduction. Despite significant differences in absolute duration, both anesthetics showed similar relative decreases in duration under acidic conditions.

DISCUSSION

Traditionally, local anesthetics' onset and duration have been attributed primarily to membrane permeability (1). However, previous research has suggested that membrane permeability alone does not fully account for clinically observed onset and duration times (17,32). Our simulations demonstrated a significant contribution of diffusion time

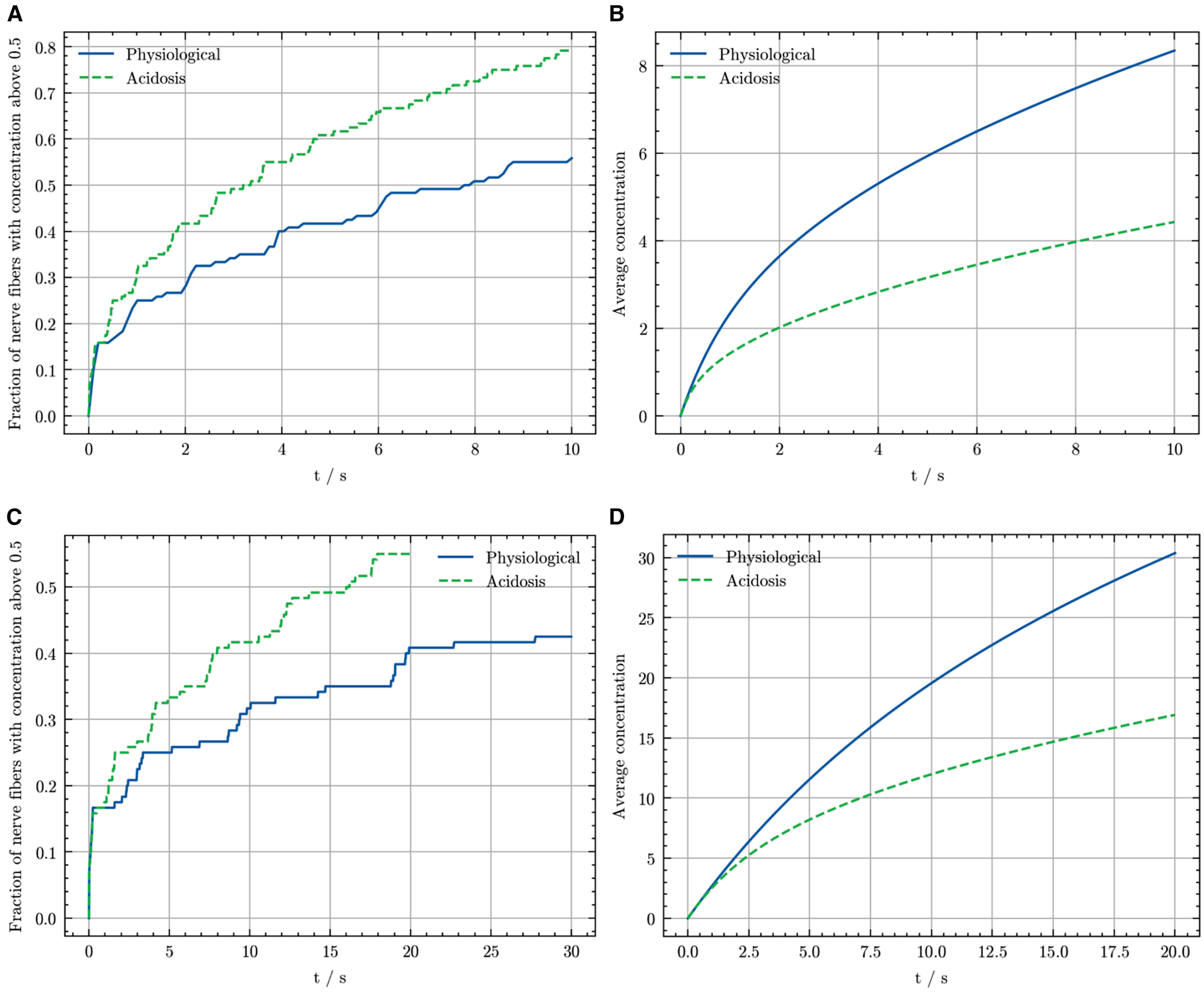

FIGURE 5 Onset kinetics for lidocaine and bupivacaine under physiological versus acidotic conditions. (*A*) Fraction of nerve fiber compartments exceeding 50% of the initial external concentration of lidocaine under physiological conditions (*blue*) and local acidosis (*dashed green*). (*B*) Average concentration of lidocaine within nerve fiber compartments over time, comparing physiological (*blue*) and acidic (*dashed green*) conditions. (*C*) Fraction of nerve fiber compartments exceeding 50% of the initial external concentration of bupivacaine under physiological (*blue*) and acidic conditions (*dashed green*). (*D*) Average concentration of bupivacaine within nerve fiber compartments over time, comparing physiological (*blue*) and acidic conditions (*dashed green*). *t* is time.

through the extracellular space within nerves to the time to onset of anesthetic action. Importantly, transmembrane diffusion time across the axolemma and Schwann cell membranes was shorter than the extracellular diffusion time. We identified an apparent slowing effect where anesthetics must first saturate peripheral compartments before effectively diffusing deeper into the fascicle. This effect intensifies with higher anesthetic octanol-water partition coefficients ($K_{o/w}$) and decreases with local acidosis. Our modeled estimates of anesthetic time durations matched clinically observed magnitudes and successfully replicated shorter durations under local acidosis and reduced equilibrium concentrations within nerve fibers, aligning well with known clinical phenomena.

The observed spatiotemporal patterns confirmed that anesthetic diffusion progressed from the periphery toward central nerve fibers. Anesthetic accumulation initially occurred predominantly in peripheral fibers, progressing inward only after these fibers approached near equilibrium concentrations exceeding the initial external concentration. These findings are consistent with earlier reports of preferential uptake in lipophilic structures (axolemma and Schwann cell layers) driven by octanol-water partition coefficients (1,15,16). Intraneuronal equilibrium concentrations are governed by extracellular-intracellular pH gradients, transmembrane potentials, and protein-binding dynamics (1,17,33). Our earlier study reported that the intraneuronal concentrations of lidocaine and bupivacaine are approximately 1.86-fold higher than the extracellular levels under physiological conditions but are reduced to 0.52 under acidic conditions (16,17). Our model confirmed lower accumulation and equilibrium concentrations during acidosis,

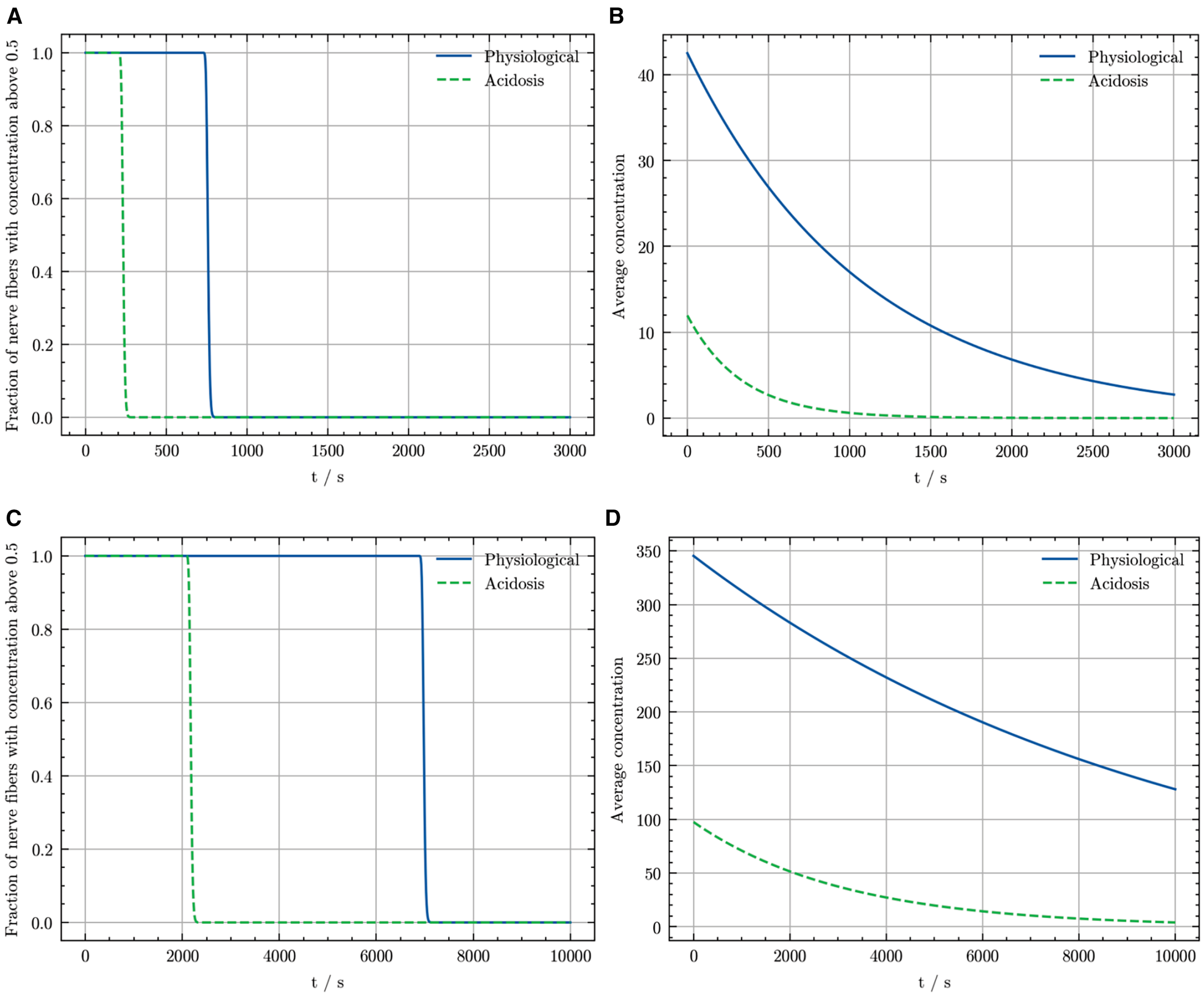

FIGURE 6 Washout kinetics for lidocaine and bupivacaine under physiological versus acidotic conditions. (*A*) Fraction of nerve fiber compartments maintaining concentrations above 50% of the initial equilibrium concentration during lidocaine washout under physiological (*blue line*) and acidic conditions (*green dashed line*). (*B*) Average concentration of lidocaine within nerve fiber compartments over time, comparing physiological (*blue line*) and acidic conditions (*green dashed line*). (*C*) Fractions of nerve fiber compartments maintaining concentrations above 50% of the initial equilibrium concentration during bupivacaine washout under physiological (*blue line*) and acidic conditions (*green dashed line*). (*D*) Average concentration of bupivacaine within nerve fiber compartments over time, comparing physiological (*blue line*) and acidic conditions (*green dashed line*).

which is consistent with clinical observations of reduced anesthetic efficacy at acidic tissue pH values (19).

Anesthetic diffusion within nerves is inherently more complex than in homogeneous media. Although lidocaine and bupivacaine diffusion coefficients are unchanged by pH, simulations indicated faster anesthetic distribution under acidic conditions, attributed to altered concentration gradients across compartments. Notably, under acidic conditions, the population of protonated LAs increases, decreasing their tendency toward lipophilic nerve compartments. Peripheral nerve compartments rapidly achieve equilibrium, accumulate LA from neighboring extracellular fluid, and thereby form temporary barriers delaying deeper diffusion. This phenomenon highlights nerve fibers acting as effective anesthetic "sinks," with transmembrane diffusion rates equal to or exceeding extracellular diffusion rates. This sink effect explains why peripheral compartments must saturate before anesthetics effectively penetrate central compartments. The effect appears correlated with $K_{o/w}$; a possible explanation is that bupivacaine yields higher concentrations at the boundary and that less LA is available for further diffusion. Despite an approximately 3.6-fold reduction in $K_{o/w}$ under acidosis, anesthetic diffusion was only 2.75-fold faster, suggesting that narrow extracellular channels facilitate rapid diffusion independent of significant $K_{o/w}$ changes.

During anesthetic washout, our results demonstrated a uniform concentration reduction across the fascicle. Capillary clearance was the limiting factor controlling washout speed, consistent with previous findings that capillary clearance rates are at least an order of magnitude slower than

compartmental transfer rates within nerve fibers (17,34). This finding clinically supports strategies involving manipulation of injection volumes and anesthetic concentrations. For example, although smaller volumes of higher concentrations may yield longer durations, they also increase potential toxicity (mainly cardiotoxicity risks), demanding precise administration (11,24,35–37).

In clinical settings, local anesthetic efficacy is markedly diminished—or even abolished—in severely inflamed tissues (18). Under local acidosis, nerve fiber storage capacity is reduced because at acidic pH, a larger proportion of LA is protonated, decreasing its affinity for the lipophilic environment. For bupivacaine, this effect is less pronounced as its protonated form still prefers 1-octanol over water (15,33). Moreover, extracellular fluid acidosis causes vasodilation, increasing perfusion and consequently LA clearance. In such cases, duration is substantially reduced or may even lead to complete absence of anesthetic effect. Notably, co-administration of vasodilators such as alcohol further exacerbates this reduction in efficacy (38). Our model explains these clinical observations.

Our fascicle model (140 μm diameter, 1254 fibers) predicted onset times of 8.7 s for lidocaine and 79.2 s for bupivacaine under physiological pH, with corresponding durations of 12.6 min and 116.3 min. Clinical studies reported somewhat longer onset and duration times (12,39), reflecting variable factors such as nerve dimensions, injection techniques, anesthetic concentrations, and vasoconstrictor inclusion (e.g., adrenaline) (1,11,13,34,39). The conservative threshold for duration estimation (defined as <50% initial equilibrium concentration in >50% fibers) further supports the underestimation of clinical durations. Under acidotic conditions, onset times are markedly shorter. Acidosis reduces the mean-force potential difference between extracellular fluid and intrafiber compartments—a change functionally equivalent to lowering fiber lipophilicity. Since transport of the lipophilic substance through the membrane is faster than that through the equivalent slab of water (17), the overall effect is a reduced mobility of LA and a reduced onset time.

Although direct comparison with clinical data remains challenging due to unknown minimal inhibitory concentrations for human nerves and by the inherently subjective nature of clinical efficacy assessments, our model closely reproduced clinically observed magnitudes without prior reliance on experimental data. The conservative threshold utilized (50% fibers at 50% concentration) aligns with known minimal inhibitory concentrations from animal studies (40), enhancing confidence in model reliability.

Nevertheless, our model has limitations, including simplification to a uniform fascicle of only Aδ-type nerve fibers, chosen due to their primary role in pain perception. Future research should incorporate nerve fiber heterogeneity (diameter, myelin thickness, and distribution) to better reflect physiological complexity. Additionally, although we assumed constant external LA concentrations, realistic clinical injections involve variable concentration gradients influenced by injection volumes, convection, and diffusion. Moreover, including a time-dependent resting potential would alter the potential of the mean force and could impact results, especially under local acidosis. Most importantly the input rate constants are only an initial estimate of the full process of diffusion of LAs into the axon compartments. The model used in the source article (17) only allowed for the estimation of the initial diffusion flux into the Schwann cell. Recent studies have shown a need for longer simulations of diffusion through Schwann cell with the Smoluchowski model or alternatively with an inhomogeneous solubility model (41), where the diffusion could actually be slower by a factor proportional the square of the number of membrane bilayers (42).

CONCLUSIONS

Our computational model effectively captured the spatiotemporal behavior of lidocaine and bupivacaine diffusion within peripheral human nerve cross sections under physiological and acidic conditions. Our findings indicate that anesthetic onset primarily depends on extracellular diffusion rather than membrane permeability alone. Consequently, the onset time should be independent of the LA pK_a. Additionally, our results confirmed that nerve fibers serve as significant LA reservoirs, with accumulation driven by lipophilicity and strongly influenced by local pH. Specifically, local acidosis substantially reduces equilibrium concentration within nerve fibers and accelerates anesthetic washout. This potentially explains the clinically observed diminished anesthetic efficacy under acidic conditions, along with contributing factors like vasodilation and increased perfusion. This reduced equilibrium concentration could fall below the critical inhibitory thresholds required for effective neuronal blockade. Although our computational model simplified the complex anatomical and biochemical variability inherent to peripheral nerves, it reliably predicted anesthetic onset and duration magnitudes consistent with clinical data. Future studies should expand this model by incorporating heterogeneous nerve fiber populations, variable nerve geometries, capillary perfusion dynamics, and the effects of time-dependent resting potential.

ACKNOWLEDGMENTS

We are grateful to Chiedozie Kenneth Ugwoke for critical review of the manuscript. The work was funded by Slovenian Research and Innovation Agency, grant nos. J3-50106 (N.U.), P3-0043 (N.U.), and P1-0012 (J.M.) and Foundation of ing. Lenarčič Milan Scholarship (V.S.).

AUTHOR CONTRIBUTIONS

V.S. contributed to conceptualization, methodology, investigation, visualization, and writing of the original draft. J.K. contributed to methodology, investigation, and visualization and reviewed and edited the manuscript. J.M. contributed to conceptualization, methodology, investigation,

supervision, and funding acquisition and reviewed and edited the manuscript. N.U. contributed to conceptualization, investigation, project administration, supervision, and funding acquisition and reviewed and edited the manuscript. All authors read and approved the final version of the manuscript.

DECLARATION OF INTERESTS

The authors declare no competing interests.

SUPPORTING MATERIAL

Supporting material can be found online at https://doi.org/10.1016/j.bpj.2025.09.026.

REFERENCES

1. Becker, D. E., and K. L. Reed. 2012. Local Anesthetics: Review of Pharmacological Considerations. *Anesth. Prog.* 59:90–101, quiz 102-3.
2. Becker, D. E., and K. L. Reed. 2006. Essentials of local anesthetic pharmacology. *Anesth. Prog.* 53:98–108, quiz 109-10.
3. Helander, E. M., A. J. Kaye, …, A. D. Kaye. 2019. Regional Nerve Blocks-Best Practice Strategies for Reduction in Complications and Comprehensive Review. *Curr. Pain Headache Rep.* 23:43.
4. Gokin, A. P., B. Philip, and G. R. Strichartz. 2001. Preferential Block of Small Myelinated Sensory and Motor Fibers by Lidocaine. *Anesthesiology.* 95:1441–1454.
5. Wang, G.-K., and G. R. Strichartz. 2012. State-Dependent Inhibition of Sodium Channels by Local Anesthetics: A 40-Year Evolution. *Biochem. Suppl. Ser. A Membr. Cell Biol.* 6:120–127.
6. Frazier, D. T., T. Narahashi, and M. Yamada. 1970. The Site of Action and Active Form of Local Anesthetics. Ii. Experiments with Quaternary Compounds. *J. Pharmacol. Exp. Therapeut.* 171:45–51.
7. Ritchie, J. M., B. Ritchie, and P. Greengard. 1965. The effect of the nerve sheath on the action of local anesthetics. *J. Pharmacol. Exp. Therapeut.* 150:160–164.
8. Högberg, C.-J., A. Maliniak, and A. P. Lyubartsev. 2007. Dynamical and structural properties of charged and uncharged lidocaine in a lipid bilayer. *Biophys. Chem.* 125:416–424.
9. Pérez-Isidoro, R., F. J. Sierra-Valdez, and J. C. Ruiz-Suárez. 2014. Anesthetic Diffusion Through Lipid Membranes Depends on the Protonation Rate. *Sci. Rep.* 4:7534.
10. Chernoff, D. M., and G. R. Strichartz. 1990. Kinetics of local anesthetic inhibition of neuronal sodium currents. pH and hydrophobicity dependence. *Biophys. J.* 58:69–81.
11. Scott, D. B., J. H. McClure, …, B. G. Covino. 1980. Effects of concentration of local anaesthetic drugs in extradural block. *Br. J. Anaesth.* 52:1033–1037.
12. Fanelli, G., A. Casati, …, G. Torri. 1998. A double-blind comparison of ropivacaine, bupivacaine, and mepivacaine during sciatic and femoral nerve blockade. *Anesth. Analg.* 87:597–600.
13. Rosenberg, P. H., E. Heinonen, …, J. Gripenberg. 1980. Differential nerve block by bupivacaine and 2-chloroprocaine. An experimental study. *Br. J. Anaesth.* 52:1183–1189.
14. Strichartz, G. 1977. Effects of tertiary local anesthetics and their quaternary derivatives on sodium channels of nerve membranes [proceedings]. *Biophys. J.* 18:353–354.
15. Kavcic, H., N. Umek, …, J. Mavri. 2021. Local Anesthetics Transfer Across the Membrane: Reproducing Octanol-Water Partition Coefficients by Solvent Reaction Field Methods. *Acta Chim. Slov.* 68:426–432.
16. Kavčič, H., N. Umek, …, J. Mavri. 2021. Local anesthetics transfer relies on pH differences and affinities toward lipophilic compartments. *J. Phys. Org. Chem.* 34:e4275.
17. Smrkolj, V., D. Pregeljc, …, J. Mavri. 2023. Micro-pharmacokinetics of lidocaine and bupivacaine transfer across a myelinated nerve fiber. *Comput. Biol. Med.* 165:107375.
18. Ueno, T., H. Tsuchiya, …, K. Takakura. 2008. Local anesthetic failure associated with inflammation: verification of the acidosis mechanism and the hypothetic participation of inflammatory peroxynitrite. *J. Inflamm. Res.* 1:41–48.
19. Boronat López, A., and M. Peñarrocha Diago. 2006. Failure of locoregional anesthesia in dental practice. Review of the literature. *Med. Oral, Patol. Oral Cirugía Bucal.* 11:E510–E513.
20. von Smoluchowski, M. 1906. Zur kinetischen Theorie der Brownschen Molekularbewegung und der Suspensionen. *Ann. Phys.* 326:756–780.
21. Kosloff, D., and R. Kosloff. 1983. A fourier method solution for the time dependent Schrödinger equation as a tool in molecular dynamics. *J. Comput. Phys.* 52:35–53.
22. Virtanen, P., R. Gommers; …, SciPy 1 0 Contributors. 2020. SciPy 1.0: fundamental algorithms for scientific computing in Python. *Nat. Methods.* 17:261–272.
23. Brill, N. A., and D. J. Tyler. 2017. Quantification of human upper extremity nerves and fascicular anatomy. *Muscle Nerve.* 56:463–471.
24. Markova, L., N. Umek, …, E. Cvetko. 2020. Neurotoxicity of bupivacaine and liposome bupivacaine after sciatic nerve block in healthy and streptozotocin-induced diabetic mice. *BMC Vet. Res.* 16:247.
25. Bento, R. F., R. Salomone, …, H. J. Z. R. Costa. 2014. Mandibular Branch of the Facial Nerve in Wistar Rats: New Experimental Model to Assess Facial Nerve Regeneration. *Int. Arch. Otorhinolaryngol.* 18:277–282.
26. Malik, R. A., A. Veves, …, A. J. Boulton. 1992. Endoneurial capillary abnormalities in mild human diabetic neuropathy. *J. Neurol. Neurosurg. Psychiatry.* 55:557–561.
27. Nestorov, I. A., L. J. Aarons, …, M. Rowland. 1998. Lumping of whole-body physiologically based pharmacokinetic models. *J. Pharmacokinet. Biopharm.* 26:21–46.
28. Appelgren, L., and D. H. Lewis. 1968. Capillary permeability-surface area product (ps) of renkin in human skeletal muscle: Preliminary report. *Acta Med. Scand.* 184:281–282.
29. Ivanov, K. P., M. K. Kalinina, and Y. I. Levkovich. 1981. Blood flow velocity in capillaries of brain and muscles and its physiological significance. *Microvasc. Res.* 22:143–155.
30. Brounéus, F., K. Karami, …, L. Sundelöf. 2001. Diffusive transport properties of some local anesthetics applicable for iontophoretic formulation of the drugs. *Int. J. Pharm.* 218:57–62.
31. Scholz, A. 2002. Mechanisms of (local) anaesthetics on voltage-gated sodium and other ion channels. *Br. J. Anaesth.* 89:52–61.
32. Istenič, S., A. Jerman, …, N. Umek. 2025. Transnasal spread of bupivacaine into the pterygopalatine fossa following endoscopically assisted cotton swab placement: a cadaveric study. *Reg. Anesth. Pain Med.* 26:rapm-2025-106553.
33. Strichartz, G. R., V. Sanchez, …, D. Martin. 1990. Fundamental properties of local anesthetics. II. Measured octanol:buffer partition coefficients and pKa values of clinically used drugs. *Anesth. Analg.* 71:158–170.
34. Almasi, R., B. Rezman, …, L. Bogar. 2020. Onset times and duration of analgesic effect of various concentrations of local anesthetic solutions in standardized volume used for brachial plexus blocks. *Heliyon.* 6:e04718.
35. Su, N., H. Wang, …, Y. Huang. 2014. Efficacy and safety of bupivacaine versus lidocaine in dental treatments: a meta-analysis of randomised controlled trials. *Int. Dent. J.* 64:34–45.
36. Hillerup, S., R. H. Jensen, and B. K. Ersbøll. 2011. Trigeminal nerve injury associated with injection of local anesthetics: needle lesion or neurotoxicity? *J. Am. Dent. Assoc.* 142:531–539.

37. Speca, S. J., S. G. Boynes, and M. A. Cuddy. 2010. Allergic reactions to local anesthetic formulations. *Dent. Clin.* 54:655–664.

38. Vlachopoulos, C., D. Tsekoura, …, C. Stefanadis. 2003. Effect of alcohol on endothelial function in healthy subjects. *Vasc. Med.* 8:263–265.

39. Costa, C. G., I. P. Tortamano, …, N. Tortamano. 2005. Onset and duration periods of articaine and lidocaine on maxillary infiltration. *Quintessence Int.* 36:197–201.

40. Yano, T., S. Ibusuki, and M. Takasaki. 2006. A Comparison of Intracellular Lidocaine and Bupivacaine Concentrations Producing Nerve Conduction Block in the Giant Axon of Crayfish In Vitro. *Anesth. Analg.* 102:1734–1738.

41. Marrink, S. J., and H. J. C. Berendsen. 1996. Permeation Process of Small Molecules across Lipid Membranes Studied by Molecular Dynamics Simulations. *J. Phys. Chem. A.* 100:16729–16738.

42. Vervust, W., S. Safaei, …, A. Ghysels. 2025. Myelin sheaths can act as compact temporary oxygen storage units as modeled by an electrical RC circuit model. *Proc. Natl. Acad. Sci.* 122:e2422437122.

Biophysical Journal, Volume *124*

Supplemental information

Spatiotemporal dynamics of local anesthetic diffusion in nerve revealed by a 2D computational model

Vladimir Smrkolj, Jakob Kralj, Janez Mavri, and Nejc Umek

Spatiotemporal dynamics of local anesthetic diffusion in nerve revealed by a 2d computational model – Supplementary information

Vladimir Smrkolj[1,2], Jakob Kralj[2], Janez Mavri[2], Nejc Umek[1]

1 Institute of Anatomy, Faculty of Medicine, University of Ljubljana, Slovenia

2 Laboratory of Computational Biochemistry and Drug Design, National Institute of Chemistry, Ljubljana, Slovenia

Derivation of the replacement coefficient

For simplicity, let's examine the transfer between two adjacent cells:

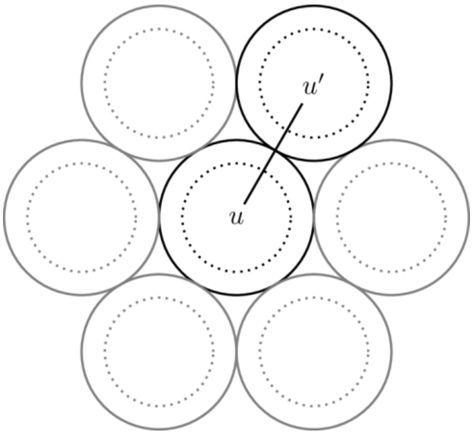

Figure S 1 Scheme of the compartment and it's soundings

Both cells have identical radius r, and may or may not be separated by a membrane. The transfer between these two compartments can be expressed as follows:

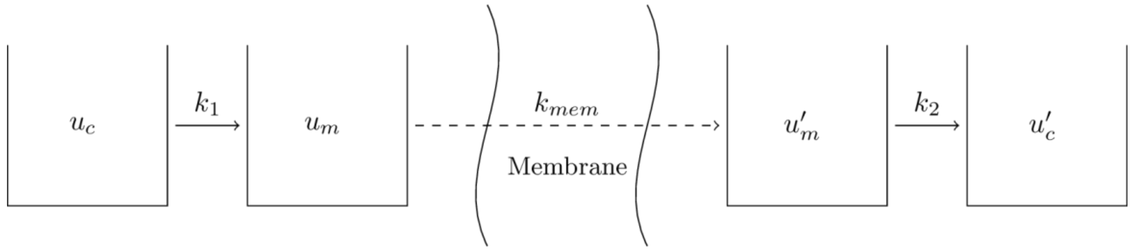

Figure S 2 Scheme of the transfer through the membrane

where u_c, u_m, u_m', u_c' represent compartments and k_1, k_2, k_{mem} denote rate constants. For the constants k_1 and k_2 we apply a standard approximation based on the diffusion coefficient D and cellular geometry:

$$k = \frac{2D}{r^2}$$

The membrane constant k_{mem} is derived from numerical simulations of the Smoluchowski PDE and varies depending on which two cells are adjacent (the rate constant differs when diffusing from inside to outside the neuron versus other directions). When diffusing between two compartments containing only inter-cellular fluid, no membrane exists, making the rate constant effectively infinite.

Considering the steady state solution, we see that the flow rate through the four compartments would be

$$J = k_1(u_c - u_m) = k_{\text{mem}}(u_m - u_m') = k_2(u_m' - u_c')$$

Let's denote a replacement constant, so that

$$J = k^*(u_c - u_c').$$

Solving the system of equation yields:

$$\frac{1}{k^*} = \frac{1}{k_1} + \frac{1}{k_2} + \frac{1}{k_{mem}}.$$

Note that where no membrane is present, $k_{mem} = \infty$ and the last term becomes zero. The combined rate constant then becomes:

$$\frac{1}{k^*} = \frac{r^2 + r'^2}{2D} + \frac{1}{k_{mem}}$$

$$\frac{1}{k^*} = \frac{k_{mem}(r^2 + r'^2) + 2D}{2Dk_{mem}}$$

This yields the final replacement coefficient:

$$k^* = \frac{2Dk_{mem}}{k_{mem}(r^2 + r'^2) + 2D}$$
